# AKT as a therapeutic target for autophagy induction and cancer therapy

**DOI:** 10.18632/oncoscience.526

**Published:** 2021-03-19

**Authors:** Qi Wu, Guido Kroemer, Oliver Kepp

**Affiliations:** ^1^Department of Breast and Thyroid Surgery, Renmin Hospital of Wuhan University, Wuhan, China; ^2^Centre de Recherche des Cordeliers, Equipe labellisée par la Ligue contre le cancer, Université de Paris, Sorbonne Université, Inserm U1138, Institut Universitaire de France, Paris, France; ^3^Metabolomics and Cell Biology Platforms, Gustave Roussy Cancer Center, Université Paris Saclay, Villejuif, France; ^4^Suzhou Institute for Systems Medicine, Chinese Academy of Medical Sciences, Suzhou, China; ^5^Pôle de Biologie, Hôpital Européen Georges Pompidou, AP-HP, Paris, France; ^6^Karolinska Institutet, Department of Women's and Children's Health, Karolinska University Hospital, Stockholm, Sweden

**Keywords:** immunotherapy, immunogenic cell death, combination therapy, flavonoids, ER stress

Successful and durable treatment of cancer requires the stimulation of adaptive immunity as well as the re-establishment of anticancer immunosurveillance. Certain antineoplastic agents, the so called immunogenic cells death (ICD) inducers, are endowed with the ability to stimulate cellular stress responses such as autophagy, transcription inhibition and endoplasmic reticulum stress, altogether leading to the liberation of danger associated molecular patterns (DAMPs) from the cancer cells. Such DAMPs include adenine nucleotide triphosphate (ATP), which serves as chemoattractant for antigen presenting cells, annexin A1 (ANXA1), which acts as a homing signal for dendritic cells, calreticulin, which functions as an phagocytosis-inducing signal and high mobility group box 1 (HMGB1), which stimulates the maturation of dendritic cells (DCs) and the presentation of tumor antigens to T cells. ICD induction is stimulated by a minority of anticancer agents such as mitoxantrone, doxorubicin and oxaliplatin that have the ability stimulate such defined patterns of immunogenic signals. However, ICD is not induced when these drugs are used at suboptimal concentrations or when drugs that are intrinsically unable to induce ICD are employed. In this latter case, ICD can be reinstated by complementing antineoplastic treatments with the pharmacological induction of cellular stress pathways such as endoplasmic reticulum (ER)-stress and autophagy [1]. Thus, a specific subset of non-toxic autophagy-inducing agents, the so called caloric restriction mimetics, such as hydroxycitrate and thiostrepton, have been shown to potentiate the release of ATP from cancer cells and to enhance anticancer immunity in mice [2, 3]. More recently, two chalcones from the chemical class of flavonoid 3,4-dimethoxychalcone (3,4-DMC) and 4,4'-dimethoxychalcone (4,4’DMC) have been identified as potent autophagy inducing agents which both differ in their mode of action. While 4,4’DMC works via the inhibition of GATA transcription factors 3,4-DMC acts through the stimulation of transcription factors EB (TFEB) and E3 (TFE3). Irrespective of this difference in their mode of action, both 3,4-DMC and 4,4’DMC enhanced anticancer immune responses in mice [4, 5].

**Figure 1:**
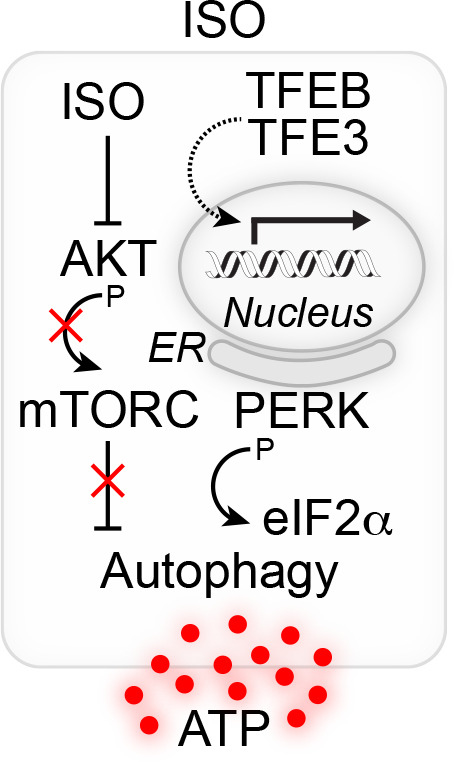
Isobacachalcone-induced anticancer immune response. **(A)**. Isobacachalcone (ISO) inhibits AKT upstream of the mechanistic target of rapamycin complex 1 (mTORC1) and triggers the activation of transcription factors EB (TFEB) and TFE3, altogether leading to the induction of autophagy. ISO further stimulates signs of the unfolded protein response (UPR) at the level of the endoplasmic retiuclum (ER) such as the PERK-dependent phosphorylation of eukaryotic initiation factor 2α (eIF2α). Both, autophagy and ER stress exhibit a certain degree of crosstalk, altogether leading to the enhanced liberation of ATP from tumor cells and the stimulation of anticancer immune responses.

Encouraged by these findings, we decided to use a system biology approach for the discovery of additional pro-autophagic agents within the chemical group of chalcones. In an unbiased screen, we identified isobacachalcone (ISO) as a non-toxic inducer of autophagic flux in vitro and in vivo in mice [6]. ISO was already known to have multiple pharmacological activities, such as anti-cancer, anti-microbial, anti-inflammatory, antioxidative as well as neuroprotective functions [1, 7, 8]. We delineated its mode of action and showed that ISO inhibits the serine/threonine kinase protein kinase B (best known as AKT) upstream of the mechanistic target of rapamycin complex 1 (mTORC1), in conjunction with an activation of TFEB and TFE3. AKT had previously been identified as therapeutic target for antineoplastic treatments as it controls mitochondrial Ca2+ homeostasis and the production of cytotoxic reactive oxygen species, thus impinging on tumor development [9, 10]. In our experiments, ISO also stimulated ER stress pathways such as the eukaryotic translation initiation factor 2 alpha kinase 3 EIF2AK3 (best known as PERK)-dependent phosphorylation of eukaryotic translation initiation factor 2α (eIF2α), and experiments on cells genetically blunted in either TF expression or eIF2α-mediated signaling pointed to a certain level of crosstalk between both pathways (Figure 1). Of note, systemic administration of ISO to tumor bearing mice stimulated anticancer immunity by enhancing the efficacy of antineoplastic treatments in an autophagy-dependent fashion [6].

In summary, our results support the notion that pharmacological induction of specific cell stress pathways has the ability to stimulate anticancer immune responses in the context of immunogenic chemotherapy. We anticipate that future investigations will yield optimized regimens for combination treatments consisting in improved ICD inducers together with additional immunotherapies such as immune checkpoint blockade. It is tempting to speculate, yet remains to be formally proven, that such combination therapies may improve the clinical efficacy of antineoplastic treatments and minimize the probability of relapse through the induction of long-lasting anticancer immune responses.
